# Predictors of Prenatal Depression: A Cross-Sectional Study in Rural Pakistan

**DOI:** 10.3389/fpsyt.2021.584287

**Published:** 2021-09-10

**Authors:** Rukhsana Khan, Ahmed Waqas, Zille Huma Mustehsan, Amna Saeed Khan, Siham Sikander, Ikhlaq Ahmad, Anam Jamil, Maria Sharif, Samina Bilal, Shafaq Zulfiqar, Amina Bibi, Atif Rahman

**Affiliations:** ^1^Department of Community Medicine Fazaia Medical College, Air University, Islamabad, Pakistan; ^2^Department of Primary Care & Mental Health, Institute of Population Health, University of Liverpool, Liverpool, United Kingdom; ^3^Human Development Research Foundation, Islamabad, Pakistan; ^4^Health Services Academy, Islamabad, Pakistan

**Keywords:** antenatal depression, prenatal depression, maternal depression, low and middle income countries, perinatal depression, Pakistan, risk factors, epidemiology

## Abstract

**Objective:** To determine the prevalence and association of prenatal depression with socioeconomic, demographic and personal factors among pregnant women living in Kallar Syedan, Rawalpindi, Pakistan.

**Methods:** Five hundred women in the second and third trimester of pregnancy, living in Kallar Syedan, a rural area of district Rawalpindi Pakistan, were included in the study. Depression was assessed using “Patient health questionnaire” (PHQ9) in Urdu, with a cut-off score of 10. Multi-dimensional scale of perceived social support (MSPSS) was used to assess perceived social support. Life Events and Difficulties Schedule (LEDS) were used to measure stressful life events in past 1 year. Tool to assess intimate partner violence (IPV) was based on WHO Multi Country Study on “Women's Health and Domestic Violence against Women.”

**Results:** Prevalence of prenatal depression was found to be 27%. Number of pregnancies was significantly associated with prenatal depression (*p* < 0.01). Women living in a joint family and those who perceived themselves as moderately satisfied or not satisfied with their life in the next 4 years were found to be depressed (*p* < 0.01, OR 6.9, CI 1.77–26.73). Depressive symptomatology in women who experienced more than five stressful life events in last 1 year was three times higher (*p* < 0.001, OR 3.2, CI 1.68–5.98) than in women with 1–2 stressful events. Women who were supported by their significant others or their family members had 0.9 times (*p* < 0.01, OR 0.9, CI 0.85–0.96) less chance of getting depressed. Pregnant women who were psychologically abused by their partners were 1.5 times more depressed (*p* < 0.05 CI 1.12–2.51). Odds of having depression was also high in women who had less mean score of MSSI (*p* < 0.05, OR 1.1, CI 1.01–1.09). Women who had suitable accommodation had 0.5 times less chance of having depression than others (*p* < 0.05, OR 0.5, CI 0.27–0.92).

**Conclusion:** Over a quarter of the women in the study population reported prenatal depression, which were predicted predominantly by psychosocial variables.

## Introduction

Pregnancy represents a vulnerable phase for women as they undergo physiological changes associated with pregnancy and prepare themselves for their new social role as a mother (1–4). Due to these changes associated with pregnancy, many women are at an increased risk of developing mental disorders especially depression. Perinatal depression can be defined as “an episode of major or minor depression with an onset either during pregnancy (**Prenatal/antenatal depression**), or during the first 12 months (**postpartum/postnatal depression**)” after delivery (5). Universally, women of child bearing age are more susceptible to develop psychopathologies with an increased risk of having depression during pregnancy (6, 7). These emotional and psychological disturbances become more pronounced in the pre- dominantly patriarchal and tribal family systems in Pakistan which are riddled with psychosocial and cultural stressors and adverse life events (8). This situation is further exacerbated by healthcare disparities in rural regions of Pakistan, where most of the births are attended by untrained midwives, resulting in high rates of complications and adverse outcomes (8).

Prenatal depression is a major public health concern in the Pakistani populace, as evident in recent literature, demonstrating a prevalence of prenatal depression of 65% in Lahore (9). According to a recent pooled analyses of 43 studies in Pakistan, the prevalence of perinatal depression is estimated at 37% during the antenatal period and 30% during the postpartum period (10). This high prevalence of prenatal depression in Pakistan has been associated with poor social network (11–13), social conflicts (12), poor economic support (14, 15), intimate partner violence, which includes psychological, physical, and sexual abuse (16), and poor dietary intake among mothers from lower socioeconomic classes (17). According to some studies, pregnancy itself can be a risk factor for intimate partner violence for women in various parts of the world (18–22). However, there are other studies which observed that when a woman suffering from intimate partner violence (IPV) becomes pregnant, the prevalence of IPV may actually become lower as compared with the previous 12 months before pregnancy (23).

In Pakistan, women reporting poor autonomy in household decisions, hailing from poor rural households, and receiving low quality and fewer years of education have shown a higher likelihood of developing prenatal depression than their counterparts (8, 24–27). In patriarchal family systems, evident in some South Asian countries, sons are preferred over daughters because they are considered to be bearers of family names, and dominant bread earners of the family. In a majority of households, birth of a daughter is associated with future concerns over expensive dowries (Urdu: *Jahaiz*) (28). In this vein, mothers giving birth to more daughters and fewer sons, are more likely to experience harassment and domestic abuse from in-laws: a strong risk factor of depression (8, 16). Previous research has also shown a weak association between having daughters and increased risk of experience of harassment and domestic abuse in Pakistani households (8).

Despite the fact that the developing world reports the highest prevalence rates for prenatal depression, most of the high quality research has been conducted in context of high income countries (29–32).

Although several studies have presented prevalence estimates on perinatal depression in Pakistan, most of these studies were limited to urban regions and hospital settings. And there is a paucity of data reported from rural settings in Pakistan (10, 29). Therefore, this study was designed to identify the risk factors of prenatal depression among rural Pakistani women.

## Methodology

### Study Design

This cross-sectional study was conducted from 1st November 2014 to 29th May 2015, in the Tehsil Kallar Syedan; district Rawalpindi of the province of Punjab, Pakistan. This area was selected because its population is ethnically and socio-economically homogenous and has primary care facilities in the area. It has accessible secondary and tertiary care services in neighboring cities, for specialized mental health care referrals. It is also the pilot site for the South Asian Hub for Advocacy, Research and Education for mental health, which aims to adapt an evidence-based intervention for perinatal depression, the Thinking Healthy Program (THP) (33–35). Ethical approval was obtained from the University of Liverpool ethics committee alongside the local “Institutional Review board of Human Development Research Foundation” (HDRF), Islamabad, Pakistan. All women gave written consent and were free to leave the interview at any time or refuse to respond to any question. They were ensured anonymity and that no individual findings would be reported. Those participants who could not read or write in the Urdu language, were explained the study objectives in detail in presence of a witness or chauffeur and provided thumb impression instead of their signatures.

**Figure 1:**
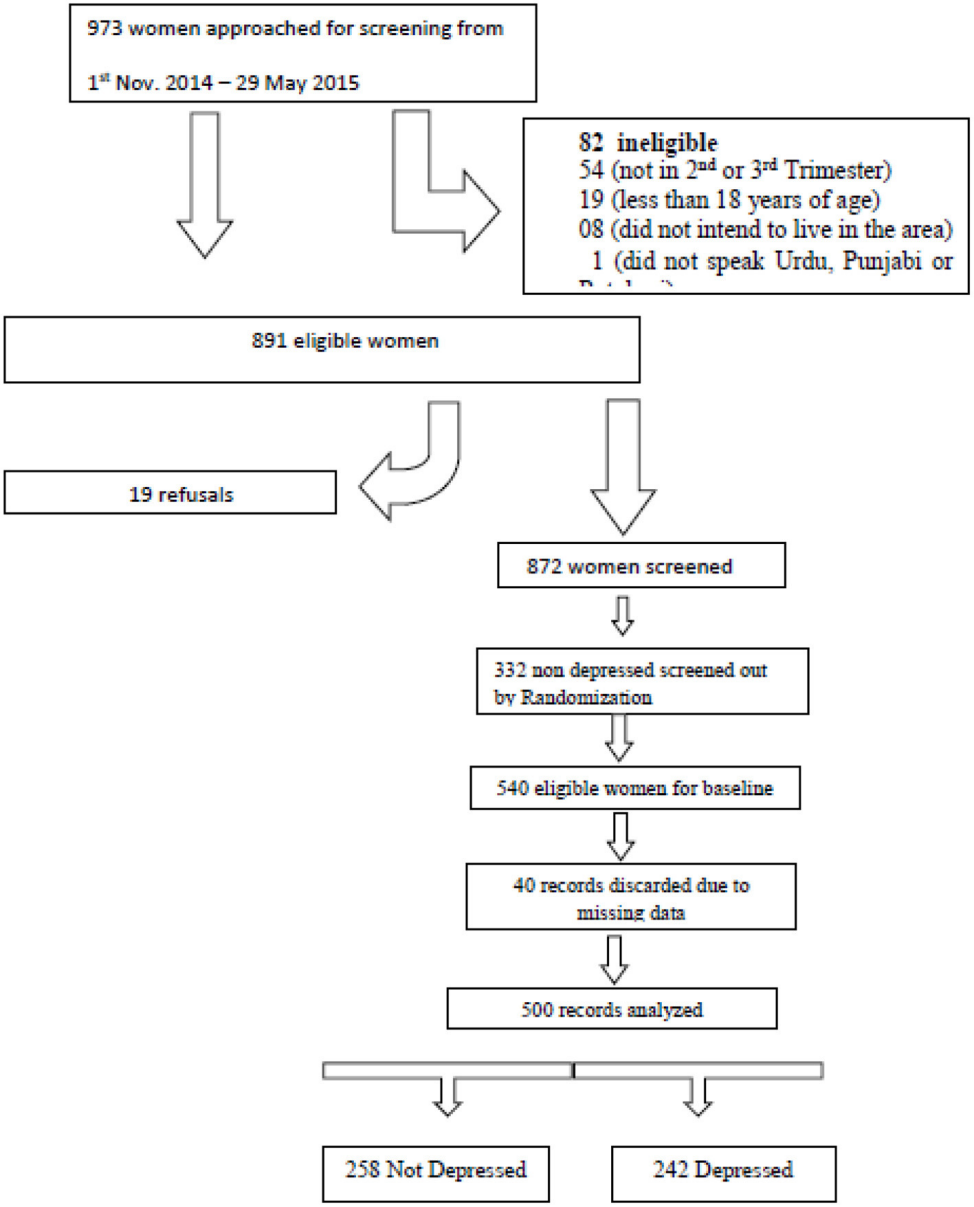
Recruitment of participants in the study.

Random sampling was employed to recruit participants in the study. The main cluster randomized controlled trial recruited participants from 40 identified clusters in the area. For inclusion in the study, we reviewed the population records maintained by the midwives and lady health workers employed at the primary care centers in Kallar Syedan. All women registered in these population records, and deemed eligible for participation, were invited to participate in the study during obstetric well-being visits conducted by the lady health workers. A total of 973 pregnant women were approached in the community for screening of depression. Out of these women, 82 women (8.43%) did not meet eligibility criteria and 19 (1.95%) refused to participate in the study, and therefore, were excluded from the study (Figure 1). Among 872 eligible women, 242 (27.75%) women reported depressive symptoms, and were recruited in this cross-sectional study. Among healthy women with no depressive symptoms, 258 out of 630 were recruited using a computer generated random number table. This subgroup was randomly selected to serve as the control group in the primary study using cRCT design; and to undergo further detailed interviews for the present study.

Although sample size calculation was done primarily for the cRCT, *post-hoc* sample size estimations are provided for this cross-sectional study. Based on previous prevalence estimates for perinatal depression of 25% in rural Rawalpindi (36), population of Kalar Syedan (*n* = 217,273,) confidence level 99% and a precision of 5%, a sample size of 497 pregnant women was required for this present study, as calculated with the following formula; *n* = z^2^*p^*^ (1-p)/d^2^: where z is the z statistic of confidence level, p is prevalence; d is the precision (37). To explore risk factors of perinatal depression, same sample size was considered suitable for regression modeling, based on the rule of thumb for estimation of sample sizes for where a minimum of 20 cases per variable need to be ensured.

### Data Collection Procedures

The Patient Health Questionnaire (PHQ-9) was used to screen the study participants for depression. The required study sample included only those who were married, in their second and third trimester of pregnancy, older than 18 years of age, spoke Urdu, Potohari, or Punjabi languages and intended to live in Kallar Sayedan for the duration of the study. Women with severe depression, suicidal tendency and psychotic illnesses were not eligible for inclusion in the study sample.

Study interviews were performed by trained graduate level psychologists, who trained and supervised by board certified psychiatrists and senior investigators in delivery of psychosocial assessment and diagnostic interviews using the Structured Clinical Interview for DSM Disorders (SCID). The survey tool was an interviewer administered questionnaire comprising of several parts: (a) demographic and socioeconomic information (b) family characteristics and autonomy in household (c) obstetric history (d) Patient Health Questionnaire (PHQ-9) (e) multi-dimensional scale of perceived social support (MSPSS) (f) Stressful life events checklist and Intimate partner violence.

The PHQ-9, translated into local languages, had been previously used in both Pakistan and India (38–40). A validated Urdu translation of the PHQ-9 was used to assess depression in the study population. Although, both the PHQ-9 and the Edinburgh Postnatal Depression Scale (EPDS) are reliable and valid scales for assessing prenatal depression (41), PHQ-9 was used in this study due to its brevity, a good internal consistency and criterion validity (39, 42). The questionnaire comprises of 9 items based on the “Diagnostic and Statistical Manual of Mental Disorders fourth edition” (*DSM*-IV) criteria. It is measured on a 4 point Likert scale ranging from “not at all” to “nearly every day” over past 14 days. These statements are summed together to reach a global measure of severity of depression. A cut-off score of 10 has shown a good sensitivity and specificity in detecting moderate levels of depression (43). Referral systems in liaison with the local community health workers, were kept in place for women who exhibited self-harm behaviors, domestic abuse or severe psychotic illnesses.

Two different scales based on heterogeneous theoretical orientations were used to measure social support in present study. Multi-dimensional scale of perceived social support (MSPSS) was developed by Zimet and colleagues and validated in a Pakistani population, exhibiting good psychometric properties (44, 45). It is a self-rating tool of perceived social support consisting of 12 questions which are rated on a 7-point scale. It yields scores on three subscales assessing social support from *Significant Other, Family and Friends*. The 7-point scale ranges from 1 “very strongly disagree” to 7 “very strongly agree.”

A previously validated scale in Pakistan, was used to assess adverse life events in the present study sample (36) based on Life Events and Difficulties Schedule (LEDS) developed by Brown and Harris (46). The use of stressful life events checklist (LEC) had been validated by some other studies as well (47–49). Degree of autonomy in participants' life was based on an elaborate checklist that assessed the frequency of household decisions in which women participated, for example, control over her earnings, visit to family and friends and care-seeking (PDHS 2012–13). Response was recorded on a scale assessing if the decision pertaining to a household event was either made by her, by husband, or other family members. These scores were then summed and categorized to full autonomy (herself only = 2), partial autonomy (herself and husband or herself and other family members = 1), and no autonomy (Husband only and others only = 0).

Tool to assess intimate partner violence (IPV) was based on WHO Multi Country Study On Women's Health And Domestic Violence Against Women (50). The items spanned across three forms of IPV including physical, sexual and psychological violence, measured by asking direct questions about the respondent's experience and frequency of specific acts.

All data was analyzed using SPSS v. 20. Quantitative variables were presented as mean (SD) and categorical variables as frequencies and percentages. The subjects identified with prenatal depressive symptoms (PHQ9 score >10) were compared with those without depressive symptoms across various socio-demographic, maternal and husband's characteristics, MSPSS, empowerment, life events, autonomy, Maternal Social Support Index (MSSI), and IPV variables using the Chi-Square test of association. Fisher's exact test was used if numbers in the cells were <5. In order to compare scores of the indices mentioned above two sample *t*-test was used.

In present study, the primary endpoint was prenatal depression recorded as a dichotomous variable; therefore, it was analyzed using Generalized Linear Model (GLM) with logit link function (logistic regression). The odds ratio between the two groups with and without depression along with 95% confidence interval were derived from the model. Several risk factors including maternal, familial, and socioeconomic status of participants, as well as different psychometric scales assessing social and spousal support, levels of autonomy, adverse life events, and intimate partner violence were introduced in the final regression model. Variance inflation factor (VIF) was used to ensure that assumption of multi-collinearity was met (51, 52). Whereas, backward regression modeling for final model selection was found to be more suitable, ensuring only significant risk factors were included in the final model. This is an iterative procedure where at each step we removed the in significant factors by using a *p*-value threshold of 0.2 and if a removed factor appeared significant in next iteration it was included. This model building process continued until a final model was achieved, containing significant risk factors of prenatal depression and an adequate goodness of fit based on likelihood ratios.

## Results

### Demographics and Maternal Characteristics

Among the 872 women undergoing screening for depression, a total of 242 (27.75%; 95% CI: 24.80–30.85%) screened positive. The final study sample analyzed for exploring risk factors, consisted of 500 pregnant women; all were married at the time of data collection, with a mean age of 26.9 (±4.80) years. Almost two thirds (63%) of our study participants were 23–30 years of age and 39.0% had at least primary education, 17.2% women had no formal education and 11.6% did graduation. These women were either in second (61%) or third trimester (39%) of pregnancy.

### Depression and Its Association With Maternal Characteristics

A total of 242 (48.4%) participants reported being depressed according to the PHQ-9. Most frequently reported symptoms were feeling of tiredness and little energy (64.4%), feeling down or hopeless (44.2%), trouble with concentration (37%), psychomotor retardation (33%), anhedonia (31.6%), hyper-somnolence/insomnia (31.4%), problems with appetite (31.4%), and poor self-esteem (22.2%). Moreover, 11.4% of the expectant mothers reported self-harming thoughts.

About half of the participants (51.2%) had normal pre-pregnancy BMI, while 23.6% of them were under weight. According to our results, 65% had <3 pregnancies and more than half of the women (55.8%) delivered their last child in hospital and reported use of contraceptive methods. Around 12.2% of the participants reported at least an infant or child death (14%) or miscarriage (28.2%) previously. Most of the participants (59.8%) had one or more than one living children. Self-reported health condition in last 30 days, was described as moderate by 39.4%, while good health by 31.8% of the expectant mothers.

Supplementary Table 1 shows the association of depression with maternal characteristics, being mother at an older age, illiteracy, having no child and bad health conditions have significant association with depression.

### Socioeconomic Characteristics and Its Association With Depression

Majority of the women were unemployed (92.4%), reported a total household income of <12,000 PKR (64.8%), with wealth index reported as poorest (20.2%), poor (39%), and average (20%). Out of total participants reported, 80% had a suitable accommodation, adequate sanitation (80.2%), and enough money to meet basic needs (80.8%) and food (78.2%). Most of the women (60.8%) women perceived that they are empowered as they could spend money at their own will, given to them by head of the household. About half of the study sample had family debts (47.2%). Maternal depression was found to be significantly associated with less income of husband, no empowerment, poor wealth index, family debt and no money for food (Supplementary Table 2).

### Family Characteristics & Perceived Autonomy

A majority of the respondents reported that their husbands were non-manual workers (60.8%), educated up to secondary school (49.6%) or primary to middle school (38.8%). Most of them lived in a joint family (63.4%) or nuclear family (22.2%) and were satisfied (57.8%) or moderately satisfied (27.2%) with their lives. Around 23.2% pregnant women perceived that they had low levels of autonomy followed by 288 (57.6%) reporting moderate levels of autonomy. Most of the study population (90.8%) did not work in last 1 year. Autonomy over different facets of women's life were explored in further details. Poorest levels of autonomy were observed over family earnings. Only 6.2% of the working women reported autonomy over their earnings. This proportion dropped drastically among unemployed participants, having husband as the sole source of household income. Exclusive control of women drops to 5% and other family members were reported having a greater control (35.6%). Healthcare decisions were mostly taken by the husband (25.8%) as compared to the women themselves (14.8%) and by both (20.4%). For major household purchases, self-decisions were reported by 16 (3.2%) and with husband 14%. Self-decisions for visit to family or relatives was reported by 40 (8.6%) and along with husband by 95 (18.8%) of the mothers (Supplementary Table 2).

### Social Support

Social support from the spouse and family was rated as inadequate in several domains. A majority of the participants (63.6%) fixed the meals themselves. House chores such as cleaning was mostly (56%) done by women themselves and only small proportion of women (18.2%) fixed the things around house, shopped for grocery (7.2%) and paid the bills (3.4%). Only 42.4% of the women reported seeking healthcare for their children themselves. Total MSSI scores were much higher among non-depressed participants (16.04 ± 6.07) than their counterparts (13.96 ± 6.02) and the difference was statistically significant (*p* < 0.001) (Table 1).

**Table 1:** Association between maternal social support Index (MSSI) and prenatal depression.

**MSSI**				**Categories**	**Depression**				***p*-value**
					**No**		**Yes**		
					***N***	**%**	***N***	**%**	
Who fix meals? Do…				You or no one	146	45.9	172	54.1	<0.001^***^
				You and someone else	23	48.9	24	51.1	
				Someone else	89	65.9	46	34.1	
Who does the grocery shopping? Do…				You or no one	15	41.7	21	58.3	0.055
				You and someone else	228	51.4	216	48.6	
				Someone else	15	75.0	5	25.0	
Who lets your children know what is right or wrong? Do…				You or no one	147	50.9	142	49.1	0.827
				You and someone else	10	47.6	11	52.4	
				Someone else	101	53.2	89	46.8	
Who fix things around the house or apartment?				You or no one	38	34.9	71	65.1	<0.001^***^
				You and someone else	164	53.4	143	46.6	
				Someone else	56	66.7	28	33.3	
Who does the cleaning?				You or no one	129	45.9	152	54.1	<0.001^***^
				You and someone else	17	39.5	26	60.5	
				Someone else	112	63.6	64	36.4	
Who pays the bills?				You or no one	8	47.1	9	52.9	0.357
				You and someone else	239	51.2	228	48.8	
				Someone else	11	68.8	5	31.3	
Who takes your children to the doctor if he/she is sick?				You or no one	114	53.8	98	46.2	0.663
				You and someone else	69	51.1	66	48.9	
				Someone else	75	49.0	78	51.0	
Who sees to it that your children go to bed?				You or no one	223	51.5	210	48.5	0.705
				You and someone else	12	60.0	8	40.0	
				Someone else	23	48.9	24	51.1	
In general, would you like to see your relatives…?				You or no one	69	49.6	70	50.4	<0.05^*^
				You and someone else	39	41.5	55	58.5	
				Someone else	150	56.2	117	43.8	
**Total MSSI score**		***N***	**MSSI score mean**	**Std. deviation**	**Mean difference**		**Confidence interval**		***p*** **-value**
Depression	No	258	16.01	6.07	2.05		0.99–3.11		<0.001^***^
	Yes	242	13.96	6.02					

* *Significant*;

*** *extremely significant*.

### Perceived Social Support

Pregnant women reporting depressive symptoms had lower MSPSS scores (35.31 ± 15.99) than their counterparts (48.88 ± 11.07). This association was found to statistically significant (mean difference = 13.56; 95% CI = 11.13–15.99; *p* < 0.001). Moreover, women with depressive symptoms perceived less support from their spouses (mean difference = 5.29; 95%CI = 4.32–6.27; *p* < 0.001), family (mean difference = 5.33; 95%CI = 4.32–6.35; *p* < 0.001) and friends (mean difference 2.93; 95% CI = 1.73–4.14; *p* < 0.001) (Supplementary Table 3).

### Stressful Life Events During the Past 1 Year

Exposure to adverse life events were reported by a high proportion of participants. Exposure to serious illness was reported by 56% of the pregnant women, financial problems (53%), problems of livelihood (52.4%), hospitalizations (44.8%), and change in social status due to marriage, divorce or start of new career (42.4%), problems related to their offspring (40.8%), and problems of residence (21.2%). Pregnant women who had experienced five or more stressful life events (56.90%) were more depressed than who had 3–4 (47.90%) or <3 (25%) as shown in Table 2.

**Table 2:** Stressful life events in past 1 year.

**S. no**	**LEC**	***N***	**%**
1	“You yourself or a closed relative of yours had been ill or had an accident which led to hospitalization”	224	44.8
2	“Any your close relative died or committed suicide or had gotten seriously ill”	281	56.2
3	“Has anyone in your family had problems of livelihood”	262	52.4
4	“You or someone in your family had any financial problems”	265	53.0
5	“You or someone in your family had changed in social status”	212	42.4
6	“You yourself have had any problem with your residence”	106	21.1
7	“Your relations with any of your close relative or friend have been troubled”	128	25.6
8	“Your marital relation with your spouse have had problem”	132	26.4
9	“You have been worried about your children's problems”	204	40.8
10	“You or other family member have had rows/quarrels amongst themselves”	124	24.8

### Intimate Partner Violence

A total 28.2% of mothers were insulted or were made to feel bad about them and 13% of women had this done to them more than 6 times. One fifth of mothers (20.4%) were both physically and sexually abused by their husbands or partners, with 18.6% reporting non-consensual sexual intercourse within 12 months, with a small proportion (8.8%) experiencing this more than 6 times. Moreover, around 18% of the women were slapped by their spouses in last 12 months, with a minority (9.2%) experiencing it 1–2 times. Overall, psychological abuse was reported by 190 (38%), physical abuse by 130 (26%) and sexual violence by 188 (37.6%) of pregnant women (Table 3).

**Table 3:** Frequencies of IPV experienced.

**Forms of violence**	**Violence ever experienced yes**		**Happened in last 12 months**		**Number of events**					
	**Yes**		**Yes**		**1–2 Times**		**3–5 Times**		**6+** **Times**	
	***N***	**%**	***N***	**%**	***N***	**%**	***N***	**%**	***N***	**%**
**IPV psychological**										
“Insulted or made her feel bad about herself”	414	82.8	122	24.4	32	6.4	25	5.0	65	13
“Did things to scare or intimidated her on purpose”	108	21.6	99	19.8	31	6.2	26	5.2	42	8.4
“To hurt her someone she care about”	31	6.2	21	4.2	6	1.2	7	1.4	8	1.6
**IPV physical**										
“Slapped/threw something/pushed/shoved”	102	20.4	90	18.0	46	9.2	22	4.4	22	4.4
“Choked or burnt on purpose”	15	3.0	12	2.4	3	0.6	4	0.8	5	1.0
“Threatened with gun /knife or other weapon”	16	3.2	13	2.6	4	0.8	4	0.8	5	1.0
“Used gun/knife or other weapon”	2	0.4	1	0.2	1	0.2	0	0.0	0	0.9
**IPV sexual**										
“Physical force to have sexual intercourse”	102	20.4	95	19.0	23	4.6	29	5.8	42	8.4
“Have sexual intercourse when did not want to”	101	20.2	93	18.6	20	4.0	29	5.8	44	8.8
“Ever force you to do something sexually that you found degrading or humiliating”	31	6.2	27	5.4	10	2.0	5	1.0	12	2.4

### Multivariable Model

Multivariable analysis no satisfaction with ones' life to be the strongest risk factor for depression (OR = 6.9; CI = 1.77–26.73; *p* < 0.01). Women reporting experiencing more than five adverse life events were three times at higher odds of having depressive symptoms than their counterparts reporting 1–2 stressful events. Participants having 4–6 pregnancies were 2.3 times at higher odds of having depressive symptoms than those who had less than 3 pregnancies. Women who were psychologically abused by their partners in last 12 months had 1.5 times more depressed (OR = 1.5; CI = 1.12–2.51; *p* < 0.05). Living in joint family was also found to be significantly associated with depression (OR = 1.9; CI = 1.06–3.55; *p* < 0.05). Women who were supported by their family, had higher MSPSS scores (OR = 0.9; CI = 0.85–0.98; *p* < 0.01), and living in a suitable accommodation exerted protective effects (Table 4).

**Table 4:** Generalized linear model for association of risk factors with prenatal depression.

**Variable**	**Categories**	**Odds ratio**	**95% CI of Odds ratio**	***p*-value**
**Maternal/obstetrics factors**	<3 (ref)			
No of pregnancies	4–6	2.3	1.33–3.91	<0.01^**^
	>6	2.2	0.74–6.53	0.159
**Family factors**				
Family structure	Nuclear (ref)			
	Joint	1.9	1.06–3.55	<0.05^*^
	Multiple	1.9	0.84–4.05	0.124
Life satisfaction in next 4 years	Satisfied (ref)			
	Moderately satisfied	2.4	1.39–4.29	<0.01^**^
	Not satisfied	6.9	1.77–26.73	<0.01^**^
Suitable accommodation	Yes	0.5	0.27–0.92	<0.05^*^
**Husband income**	<12,000 (ref)			
	12,001–21,000	0.8	0.46–1.27	0.29
	21,000–30,000	1.4	0.60–3.24	0.44
	>30,001	0.4	0.17–1.15	0.09
**LEC score**	1–2 (ref)			
	3–4	1.7	0.86–3.38	0.12
	5+	3.2	1.68–5.98	<0.001^***^
**IPV psychological**	Yes	1.5	1.12–2.51	<0.05^*^
**Maternal perceived social support (MSPSS)**	Family	0.9	0.89–0.98	<0.01^**^
**Maternal social support index (MSSI)**		1.1	1.01–1.09	<0.05^*^

* *Significant*;

** *highly significant*;

*** *extremely significant*.

## Discussion

This study reveals a significant prevalence of prenatal depression in a rural setting in Pakistan. This burden of depression among pregnant women is predicted by the unique sociocultural environment of Pakistan. A high number of study population reported poor autonomy and empowerment in their daily lives, inadequate social support, and a high frequency of intimate partner violence and adverse life events. A negative correlation was found between nuclear family structure, economic prosperity and prenatal depression.

The prevalence of prenatal depression in the study population was estimated to be around 27%, which is significantly higher than the pooled prevalence of 15.6% in low and middle income countries (29, 53). These statistics are in consonance recent meta-analytical estimates for perinatal depression reported by Atif et al. (10). The range of prevalence of prenatal depression in Pakistan, is reported from a minimum of 11.5–75% (9, 27, 54–59). However, these studies differ in their study setting, screening instruments, and heterogeneous ethnicities and cultural backgrounds of participants. These figures are unequivocally higher than those reported in high income developed countries, for instance, 8.7% in Hong Kong (using BDI score 15) and 6.1% in USA (60).

Only 57.8% of the participants reported satisfaction with their lifestyle- the strongest risk factor of prenatal depression. This finding has been replicated among Jordanian women, exhibiting moderate to strong association between prenatal depression and rates of satisfaction (61). The level of satisfaction with life and psychiatric symptoms may be driven by increased resilience in face of adversity among our study sample, keeping in mind the religious and cultural norms followed by the people of the sub-continent.

In several LMIC settings, leaving a marriage is not an option for married women even in the severe adversity such as spousal violence. Obedience to husbands is overtly emphasized to preserve the patriarchal fabric of the rural Pakistani culture and these factors sometimes lead to a higher prevalence of physical punishment rendered to wives or daughter in laws. Physical violence, although, not encouraged in rural Pakistan, may have some acceptability as presented in this study. A high prevalence of intimate partner violence has been reported in neighboring regions as well. For instance, prevalence of physical violence by husbands is reported to be 35.9% in Maharashtra, India (62), and psychological IPV to be 75.9% in a multi-hospital based study in Pakistan (63). Nevertheless, exposure to all the three forms of violence may increase the risk of prenatal depression.

In a similar vein, over half of the participants reported experiencing some form of adverse life event that is in consonance with another study conducted in a rural sub district of Rawalpindi by Rahman and colleagues (36). Previous research has established that despite the cultural norms, pregnancy itself is a great stressor when a woman is transitioning into her new social role as a mother. This social stress is further exacerbated by physiological changes that accompany pregnancy. The aforementioned factors including poor access to obstetric healthcare, socioeconomic disparities as well as superimposed exposure to adverse life events may trigger prenatal depression (6, 64).

Social support is an important risk factor of depression and has demonstrated cross-cultural significance (65–67). Studies carried out in Karachi, Pakistan also observed that the presence or absence of social support could forecast the occurrence of prenatal depression (68, 69). In our study sample, low scores on all three subscales of social support from family, friends and spouse were recorded. However, only family support was found to be significant in multivariable model. Women who were supported by their family had less chance of getting depressed than those who had no support. Contrary to our findings, Husain et al. reported that none of these subscales predicted prenatal depression among British-Pakistani women (70).

Surprisingly, we observed that women living in with in-laws in a joint family system, reported higher odds for prenatal depression. This finding seems contrary to our previous finding for social support. It should be noted that the MSPSS utilized in our study, assesses the quality of perceived social support from family members including women in laws and biological family. Perhaps this could be explained by the preference of women to live in a nuclear family with their own children and husband. Living in a nuclear family may lead to more autonomy in a woman's life and also reduce extra responsibilities of other family members (71). This finding was however, not substantiated by the study carried out by Rahman et al. where they identified joint family as a protective factor for depression (36). Living in a joint family structure is considered more acceptable in rural Pakistan, in contrast to higher income countries or major cities of Pakistan such as Lahore, where nuclear family structures are preferred.

Perhaps this could be looked upon from another angle. Pregnant women who receive some sort of tangible assistance in rearing of their children and household chores from their family members (other than spouse), may report higher satisfaction living in joint families. This is corroborated by our analyses related to lower frequency of depression among women who received tangible assistance from someone other than their spouses according to the MSSI scale.

## Strengths and Limitations

This study has several strengths. This study is one of the few epidemiological studies conducted in a rural setting in Pakistan. Other strengths include an adequate sample size that was randomly sampled to achieve a representative study sample. Despite of its strengths, it also has a few limitations. Its restriction to one study site may limit generalizability to entire Pakistani population. Due to cultural factors, information regarding sensitive variables such as domestic violence and harassment may have been reported by the participants. A cross-sectional study design limits inference related to causality and use of self-report measures may introduce recall bias in the study. Both the Maternal Social Support Index (MSSI) and the WHO tool for assessment of Intimate Partner Violence are well-established constructs; however, no validation study has yet been published to ascertain their construct validity and reliability for use in Pakistan.

## Conclusion

The study shows that over a quarter of all pregnant women in second or third trimester show evidence of depression. Attention is needed to develop cost-effective intervention strategies. The families that are in transition from a more traditional way of life to the one which is influenced by recent socio-demographic and economic changes are more vulnerable. Satisfaction with life, family structure, adequate housing, social support, economic well-being, and intimate partner violence were all risk factors of prenatal depression in rural Pakistan.

## Data Availability Statement

The raw data supporting the conclusions of this article will be made available by the authors, without undue reservation.

## Ethics Statement

The studies involving human participants were reviewed and approved by University of Liverpool, Liverpool, UK. The patients/participants provided their written informed consent to participate in this study.

## Disclosure

This study was submitted for partial fulfillment for Doctor of Philosophy in Public Health at the University of Liverpool, UK.

## Author Contributions

RK made major contributions in the concept and design of the study, analysis and interpretation of data, and manuscript writing. AW contributed in study design and manuscript writing. IA, AJ, MS, SB, SZ, and AB collected the data and performed field work. AB and ZM participated in draft writing. AR and SS supervised the project, guidelines, and intellectual review of manuscript at every stage. All authors approved the final draft of the manuscript.

## Conflict of Interest

The authors declare that the research was conducted in the absence of any commercial or financial relationships that could be construed as a potential conflict of interest.

## Publisher's Note

All claims expressed in this article are solely those of the authors and do not necessarily represent those of their affiliated organizations, or those of the publisher, the editors and the reviewers. Any product that may be evaluated in this article, or claim that may be made by its manufacturer, is not guaranteed or endorsed by the publisher.
