# Supplementary material for: Predictors of Prenatal Depression: A Cross-Sectional Study in Rural Pakistan
Source: Front Psychiatry. 2021 Sep 10;12:584287. doi: 10.3389/fpsyt.2021.584287 (PMC8461022; doi:10.3389/fpsyt.2021.584287)
Supplement: Supplementary file 1 [file Data_Sheet_1.pdf]

Supplementary table 1: Association of maternal characteristics with prenatal depression

Maternal /Obstetrics characteristics	Categories	Depression				p-value
		No		Yes		
**Mean age in years**			26.5 ±4.3		27.3± 5.2	<0.05*
**Mean BMI**			26.1 ±15.3		25.1 ±8.9	0.403
		**N**	**%**	**N**	**%**	
**Age category**	16-22<br>23-30<br>31+	49<br>171<br>38	53.8<br>53.9<br>41.3	42<br>146<br>54	46.2<br>46.1<br>58.7	0.091
**women's education**<br>No formal education<br>Primary/Middle<br>Sec/H. secondary<br>Bachelors		 33<br>87<br>99<br>39	 38.4<br>44.6<br>61.5<br>67.2	 53<br>108<br>62<br>19	 61.6<br>55.4<br>38.5<br>32.2	<0.001***
**BMI (Pre-Pregnancy)**<br>Under weight<br>Normal weight<br>Overweight<br>Obese		 65<br>130<br>51<br>12	 55.1<br>50.8<br>51.0<br>46.2	 53<br>126<br>49<br>14	 44.9<br>49.2<br>49.0<br>53.8	0.810
**Infant Death (under one year Death)**	No<br>Yes	231<br>27	52.6<br>44.3	208<br>34	47.4<br>55.7	0.221
**Child mortality (1-5 year Child Death)**	No<br>Yes	226<br>32	52.3<br>47.1	206<br>36	47.2<br>52.9	0.420
**Miscarriage**	No<br>Yes	195<br>63	54.3<br>44.7	164<br>78	45.7<br>55.3	0.052
**No of living children**	0<br>1-3<br>4+	46<br>187<br>25	30.6<br>62.7<br>49.5	104<br>112<br>26	69.3<br>37.3<br>50.5	<0.001***
**Duration of pregnancy**	2nd trimester<br>3rd trimester	96<br>162	31.4<br>83.1	209<br>33	68.6<br>16.9	0.631
**Health in last 30 days**	Good<br>Moderate<br>Bad	144<br>101<br>13	90.5<br>51.2<br>9.0	15<br>96<br>131	9.43<br>48.7<br>91.0	<0.001***
**Delivery place**	Hospital<br>Home<br>other	161<br>35<br>62	57.7<br>48.4<br>41.7	118<br>37<br>87	42.3<br>51.6<br>58.3	<0.05*
**Plan to use Modern Contraceptives**	No<br>Yes	138<br>120	62.4<br>43.0	83<br>159	37.6<br>57.0	0.151
**No of pregnancies**	< 3<br>4-6<br>> 6	158<br>87<br>13	49.1<br>59.2<br>41.0	164<br>60<br>18	50.9<br>40.8<br>59.0	<0.001***

*Significant **highly significant ***extremely significant

Supplementary Table 2 Socioeconomic characteristics and depression

Socioeconomic Factors	Categories	Depression				p-value
		No		Yes		
		N	%	N	%	
Women employment	No	242	52.4	220	47.6	0.223
	Yes	16	42.1	22	57.9	
Husbands income	< 12,000	134	45.7	159	54.3	<0.01**
	12,001-21,000	83	59.3	57	40.7	
	21,001 – 30,000	18	52.9	16	47.1	
	>30,001	23	69.7	10	30.3	
Total income	< 12,000	161	49.7	163	50.3	0.480
	12,001-21,000	31	52.5	28	47.5	
	21,001 – 30,000	24	51.1	23	48.9	
	>30,001	42	60.0	28	40.0	
Women empowerment status	No	69	35.2	127	64.8	<0.001***
	Yes	189	62.2	115	37.8	
Wealth index	Poorest	27	26.7	74	73.3	<0.001***
	Poor	92	47.2	103	52.8	
	Average	63	63.0	37	37.0	
	Rich	61	72.6	23	27.4	
	Richest	15	75.0	5	25.0	
Have money for basic needs	No	21	23.3	69	76.7	<0.001***
	Yes	236	58.4	168	41.6	
	Don’t know	1	16.7	5	83.3	
Money for food	No	23	25.3	68	74.7	<0.001***
	Yes	226	57.8	165	42.2	
	Don’t know	9	50.0	9	50.9	
Family debt	No	150	64.9	81	35.5	<0.001***
	Yes	88	37.3	148	62.7	
	Don’t know	20	60.6	13	19.4	

**Family structure**	Nuclear<br>Joint<br>Multiple	56<br>173<br>29	50.5<br>54.6<br>40.3	55<br>144<br>43	49.5<br>45.4<br>59.7	0.087
**Husband away from home in last 6 months**	No<br>Yes	206<br>52	50.6<br>55.9	201<br>41	49.4<br>44.1	0.356
**Life satisfaction**	Satisfied<br>Moderately<br>Not satisfied	210<br>42<br>6	72.7<br>30.9<br>8.0	79<br>94<br>69	27.3<br>69.1<br>92.0	<0.001***
**Expectation for life satisfaction in next four years**	Satisfied<br>Moderately<br>Not satisfied	224<br>31<br>3	65.9<br>27.7<br>6.3	116<br>81<br>45	64.1<br>72.3<br>93.8	<0.001***

*Significant **highly significant ***extremely significant

Supplementary Table 3: MSPSS scores and prenatal depression

	Depression Yes	Depression No			
**MSPSS score**	**Mean SD**	**Mean SD**	**mean difference**	**CI**	**p-value**
Significant others subscale	**18.43(3.86**	**13.14(6.75)**	**5.29**	**4.32-6.27**	<0.001
Family subscale	**18.9 (4.02)**	**12.85(6.99)**	**5.33**	**4.32-6.35**	<0.001
Friends subscale	**12.26(7.17)**	**9.32(6.58)**	**2.93**	**1.73-4.14**	<0.001
**MSPSS total**	**48.88(11.07)**	**35.31(15.99)**	**13.56**	**11.13-15.99**	<0.00
